# Supplementary material for: Exploring the evolution and function of Canoe’s intrinsically disordered region in linking cell-cell junctions to the cytoskeleton during embryonic morphogenesis
Source: PLoS One. 2023 Aug 3;18(8):e0289224. doi: 10.1371/journal.pone.0289224 (PMC10399776; doi:10.1371/journal.pone.0289224)
Supplement: Attachment — Submitted filename: ResponseToReviews.pdf [file pone.0289224.s001.pdf]

Response to reviews: PONE-D-23-06606

We are grateful to the reviewers for their generally positive response and thoughtful suggestions regarding our manuscript. We have followed their suggestions, addressing all of the issues by adding new data and making the suggested changes in the text and Figures. We carried out the suggested analysis of heterozygous effects on viability suggested by Reviewer 1, added the stacked graph displays of phenotypes suggested by Reviewer 3, and revised the text and Figure presentation as suggested. Figure 7,8,9,10,11,12,13,and 14 were all revised, following the suggestions of the reviewers. We attached a marked-up version of the text with the changes highlighted in red. These revisions strengthened and extended our work and we are grateful for their suggestions. Our responses to each point are below in italics.

Reviewers' comments:

Reviewer's Responses to Questions

Comments to the Author

1. Is the manuscript technically sound, and do the data support the conclusions?

The manuscript must describe a technically sound piece of scientific research with data that supports the conclusions. Experiments must have been conducted rigorously, with appropriate controls, replication, and sample sizes. The conclusions must be drawn appropriately based on the data presented.

Reviewer #1: Yes

Reviewer #2: Yes

Reviewer #3: Yes

---

2. Has the statistical analysis been performed appropriately and rigorously?

Reviewer #1: Yes

Reviewer #2: Yes

Reviewer #3: Yes

---

3. Have the authors made all data underlying the findings in their manuscript fully available?

The PLOS Data policy requires authors to make all data underlying the findings described in their manuscript fully available without restriction, with rare exception (please refer to the Data Availability Statement in the manuscript PDF file). The data should be provided as part of the manuscript or its supporting information, or deposited to a public repository. For example, in

addition to summary statistics, the data points behind means, medians and variance measures should be available. If there are restrictions on publicly sharing data—e.g. participant privacy or use of data from a third party—those must be specified.

Reviewer #1: Yes

Reviewer #2: Yes

Reviewer #3: Yes

---

4. Is the manuscript presented in an intelligible fashion and written in standard English?

PLOS ONE does not copyedit accepted manuscripts, so the language in submitted articles must be clear, correct, and unambiguous. Any typographical or grammatical errors should be corrected at revision, so please note any specific errors here.

Reviewer #1: Yes

Reviewer #2: Yes

Reviewer #3: Yes

---

5. Review Comments to the Author

Please use the space provided to explain your answers to the questions above. You may also include additional comments for the author, including concerns about dual publication, research ethics, or publication ethics. (Please upload your review as an attachment if it exceeds 20,000 characters)

Reviewer comments are included in their entirety, and Responses are in italics

Reviewer #1: This manuscript attempts to identify the role of IDRs in Canoe and Afadin by performing an indepth structure analysis along with experiments of mutants in vivo. The work in the manuscript is carried out in a rigorous manner with good controls and enough n values along with the relevant statistics. I recommend the manuscript for publication with a few minor additions suggested below.

The authors find that the IDRs diverged more rapidly as compared to the well defined domains.

One caveat in the study is whether the truncated canoe protein is expressed in the mutants and that was not ascertainable due to the absence of a suitable antibody.

In general the analysis of the different mutants is done well, just one analysis which would have been nice to add is the lethality of the heterozygous mutants themselves and an analysis of heteroallelic combinations to further emphasize the complementation between domains of the

mutants. This is not essential but a discussion on this complementation will help understand the function of different domains. This will also help impart an important function to the IDRs.

We are grateful for this favorable assessment and thoughtful suggestions. We have made sure the important caveat you note is clearly stated in the Discussion.

Since the orientation of the embryos is different directions, it is hard to decipher the phenotypes being described in the figures. I request the authors to as far as possible choose sample images in the same orientation.

Our apologies for our lack of clarity in this regard, giving us the opportunity to clarify image orientation throughout. It is the convention in our field to orient immunofluorescence images of embryos anterior to the left, and, unless a different view is needed, oriented dorsal up. We have 1) verified that all of our images fit this convention 2) clearly labeled the first Figure where we show these images, and 3) Edited each of the Figure legends to explicitly state the orientation. Our labs practice and that of many of our colleagues for cuticle images is different—these are oriented anterior up, to facilitate comparison between wildtype and mutants. For cuticles we have also clearly labeled the first Figure where we show these images, and edited each of the relevant Figure legends to explicitly state the orientation.

Is it possible that the severe defect seen in the canoe mutants in the IDR domain occurs due to partial dominant negative defects. Heterozygous allele embryonic lethality at different temperatures would have been useful to assess this effect.

This was a good suggestion and we have carried out the suggested experiment. We out crossed females from four alleles Balanced over the TM3 Balancer to wildtype males, and counted Balanced and unbalanced progeny. We carried this out at 18 degrees and 25 degrees. The four alleles we used were: 1) cno^{R2} [our canonical null allele with a early stop codon and a "weak" phenotype, 2+3) cno^{2} and cno^{R17}, two alleles with stop codons near the beginning of the IDR and the two alleles with the strongest phenotypes 4) cno^{R8}, our only missense allele and the allele with the weakest phenotype. The data are below.

	Relative viability versus Balancer Chromosome	
	18°	25°
cnoR2/TM3 x WT	54%	53%
cno2/TM3 x WT	54%	49%
cnoR17/TM3 x WT	51%	54%
cnoR8/TM3 x WT	49%	57%

If these strong alleles had a strong dominant-negative effect, we predicted that their heterozygous viability would be lower than that of the null and potentially that of the weak allele cno^{R8}. However, as you will see, there were no consistent differences suggesting strong effects of the two alleles with truncations early in the IDR. At 18 degrees cno^{R8} had the lowest viability and cno^{2} viability was identical to that of cno^{R2}. At 25 degrees, while cno^{2} viability was lower, the viability of cno^{R17} was higher than that of the null allele. Thus, none of the alleles has a strong dominant-negative effect—we thus suspect the effects we see are due to the very low levels of

maternal zygotic Cno protein late in embryonic development, making the phenotype susceptible to subtle disruptions of maternal protein function. To fully define whether proteins truncated in the IDR are dominant negative, it will be important to engineer alleles that produce truncated proteins without the caveat of early stop codons, as we did with cnoΔFAB, to test this hypothesis. We now have added this data and the accompanying text to the relevant section of the Discussion.

Also is it known if these mutants can all be rescued by addition of a duplication of the locus or a transgene with the endogenous promoter? This will help delineate the impact of off target mutations in the alleles on the phenotypes.

Given the number of alleles involved, this would be a very substantial amount of work. Instead, we have noted the caveat as follows: : "In the future, it would be useful to ensure that each allele can be rescued by a transgene carrying wildtype cno, to ensure that other mutations on each chromosome are not affecting the phenotype." We did ensure that the lethality of all alleles was due to a lesion in cno by verifying that all are lethal over the null allele, cno^{R2}. We also now clarify that most of the alleles were generated on the same genetic background, and added the following caveat

Reviewer #2: In this manuscript, the authors have extensively investigated the Drosophila Canoe protein and its vertebrate ortholog Afadin using bioinformatic and genetic analyses. The paper is well written and the data are well presented.

We are grateful for this favorable assessment.

Reviewer #3: Gurley et al. investigated the evolutional conservation and function of the intrinsically disordered region (IDR) of Canoe/Afadin in this paper. Using bioinformatics, they found that each of Canoe and Afadin has multiple sequence motifs that are conserved over shorter evolutionary periods. Analysis of zygotic and maternal/zygotic mutants of canoe suggested that IDR is important for Canoe's protein function. These results shed new light on a relatively unexplored region of Canoe/Afadin and provide a new starting point of future research.

We are grateful for this favorable assessment and thoughtful suggestions.

Minor points and suggestions
1. Discussion pp. 37-38, lines. 850-865
The logic supporting the conclusion that IDR is important for Canoe function is not easy to follow. This section of the Discussion (and related part) should be rewritten so that the conclusion is convincingly acceptable.
My understanding is as follows:
Zygotic mutant data indicate that prematurely truncated proteins have a dominant-negative effect, suggesting that IDR-truncated proteins may escape from nonsense-mediate mRNA decay in the zygotic mutants. This suggests that these molecules are also expressed in the maternal/zygotic mutants but the mutants exhibit a severe phenotype comparable to that of the null mutant, strongly suggesting an essential role of IDR.

Thank you for this very accurate assessment and concrete suggestion. In attempting to avoid making unwarranted conclusions, we had made this section so convoluted that our major points and their caveats were lost. We have completely revised this section of the Discussion, putting a summary like that above at the beginning of the section, and then shortening and clarifying the

more detailed discussion that follows.

2. Figs. 9G-J, 10B, 12I-N, 14A-B
It is difficult to compare differences between mutants in phenotypic severity at a glance. I would suggest that the authors use a stacked vertical bar chart (100% cumulative bar chart).

This was an excellent suggestion—we have added the requested charts of each Figure.

3. Fig. 6I
Using AlphaFold and AlphaFold-Multimer, it is predicted that the conserved region of Afadin (1403-1455) forms an alpha-helix and interacts with alpha-E-catenin M3. Since alpha-E-catenin is an important tension transducer of adherens junctions, it would be useful to discuss whether its interaction with Afadin affects its conformation, dimerization state, and binding to other alpha-E-catenin-binding partners (such as beta-catenin, vinculin, and F-actin).

This is a good point—we have added mention of this to the revised Discussion, as follows: "Since alpha-catenin is a critical player in sensing and transducing tension at AJs [2], it will be important in the future to test whether its interaction with Afadin affects its conformation, dimerization state, and binding to other alpha-catenin-binding partners, such as beta-catenin, vinculin, and F-actin. Mutational analysis of this motif in cultured mammalian cells would be one route forward."

Prompted by this suggestion we also have added further discussion of the potential for interaction between Drosophila Canoe and alpha-catenin later in that section of the Discussion, as follows: "In fact, there are no long conserved motifs N-terminal to the two predicted alpha-helices that comprise the potential F-actin binding site in Drosophila. However, mutations altering the M-region of Drosophila alpha-catenin alter the recruitment of Cno to tricellular junctions [55], consistent with some sort of interaction, direct or indirect. Thus, it will be important to determine if Drosophila Cno directly interacts with alpha-catenin, and to define the mechanisms by which alpha-catenin regulates Cno recruitment to AJs under tension."

Typographical error
p. 26, l.582-583 seems to be repetitive.
"comparing one of our putative null alleles with an early stop codon, cnoR10, with one of our alleles with the alleles with the strong phenotype, cnoR17."

Fixed—thanks. It now reads: "We furthered this analysis of the zygotic phenotypes by comparing the effects on cell shapes and morphogenesis on embryos during and after dorsal closure in one of our putative null alleles with an early stop codon, cno^{R10}, with one of our alleles with the alleles with the strong phenotype, cno^{R17}."
